# Enterovirus 71 infection induces pyroptotic brain injury via synergistic activation of classical inflammasome and viral gasdermin D cleavage

**DOI:** 10.1128/jvi.01860-25

**Published:** 2025-11-25

**Authors:** Tianrun Liu, Baixin Wang, Yingyu Li, Shuming Tian, Xinlong Gao, Yuhan Fan, Xiaomeng Zhang, Limin Yang, Rui Wu, Lei Liu

**Affiliations:** 1 School of Medicine, Jiamusi University 470003, Jiamusi, China; 2 The First Affiliated Hospital of Jiamusi University, Jiamusi, China; 3 School of Medicine, Dalian University 74547, Dalian, China; St Jude Children's Research Hospital, Memphis, Tennessee, USA

**Keywords:** Enterovirus 71, pyroptosis, inflammasome

## Abstract

Enterovirus 71 (EV71) is a viral pathogen responsible for causing hand, foot, and mouth disease, which can lead to severe neurological complications. This study aims to elucidate the pyroptosis mechanism underlying brain injury induced by EV71 infection. EV71-infected BALB/c suckling mice exhibited characteristic symptoms, including weight loss, lethargy, and limb weakness. Notably, elevated levels of the inflammatory proteins IL-1β and IL-18 were detected in the brain tissue of the infected mice. Research findings indicate that EV71 infection activates the NLRP3 inflammasome, resulting in an increased release of IL-1β and IL-18. Furthermore, upregulation of the expression of Caspase-1, Caspase-11, and the pyroptosis-related protein GSDMD was observed in the context of EV71 infection. Importantly, the administration of inhibitors targeting Caspase-1 and Caspase-11 led to the downregulation of these protein expression levels and simultaneously reduced the severity of the inflammatory response in the brain tissue. These results highlight the critical regulatory role and cross-talk between Caspase-1 and Caspase-11 in EV71-induced brain injury, which involves inflammatory responses and pyroptosis. The significance of these findings enhances our understanding of the pathogenic mechanisms associated with EV71 infection and offers valuable insights for the development of new therapeutic strategies.

**IMPORTANCE:**

This study elucidated the molecular mechanism underlying pyroptosis-mediated brain injury during EV71 infection in hand, foot, and mouth disease (HFMD), addressing a critical knowledge gap in neuroinflammatory pathogenesis. Using a BALB/c suckling mouse model, we demonstrated that EV71 infection induced a significant upregulation of the pro-inflammatory cytokines IL-1β and IL-18 in brain tissues. Mechanistically, the activation of the caspase-1/11-GSDMD axis was confirmed via Western blot analysis, which revealed an increase in cleaved GSDMD levels in the presence of EV71, indicating a definitive link between the virus and pyroptotic cell death, as supported by studies on GSDME’s role in EV71-induced cell pyroptosis. Specific inhibitors targeting caspase-1/11 have been shown to effectively suppress protein expression, reduce neuroinflammatory markers, and improve survival rates, as demonstrated in studies involving acute pancreatitis, EAE, and non-canonical cell death. These findings not only advance the understanding of EV71 neuropathogenesis but also identify caspase-1/11 as promising therapeutic targets for mitigating HFMD-associated brain injury.

## INTRODUCTION

*Enterovirus* 71 (EV71), a member of the Enterovirus genus within the *Picornaviridae* family, is a single-stranded positive-sense RNA virus with a genome length of approximately 7.5 kb (1). As a significant human pathogen, EV71 is well recognized as a major etiological agent of hand, foot, and mouth disease (HFMD). While most HFMD cases caused by EV71 follow a self-limiting clinical course, severe complications (such as neurological disorders) can occur in some instances, highlighting its clinical relevance (2). However, it is crucial not to underestimate the potential for severe neurological complications linked to EV71 infection. The virus spreads through gastrointestinal and respiratory routes as well as via direct contact (3). EV71 is identified as the primary virus triggering neurological complications, such as neurogenic pulmonary edema, brainstem encephalitis, aseptic meningitis, and acute flaccid paralysis, particularly threatening the health of children (4, 5). Currently, no specific drug or clinically proven effective treatment for HFMD caused by EV71 exists. Commonly employed treatment methods include symptomatic approaches, such as glucocorticoids and antibiotics (6).

Understanding the dynamics of the inflammatory response during EV71 infection is crucial for effective treatment. In the early stages of EV71 infection, the host initiates pyroptosis via an inflammatory response, a process that inhibits virus replication by activating Gasdermin D (GSDMD). Cleavage of GSDMD by Caspase-1 dimers following inflammasome activation increases cell membrane permeability, leading to cell pyroptosis—an event that not only disrupts the virus’s replication site (as cell lysis inherently impairs viral replication niches) but also directly lowers progeny virus yield due to the loss of viable host cells required for viral propagation (7–9). However, as the EV71 infection progresses, the inflammatory-triggered pyroptosis can overwhelm the host’s compensatory capacity, causing damage to the host’s tissues and organs. In severe cases, it can lead to death. This parallels the pattern seen in conditions like COVID-19, where an excessive inflammatory response can lead to acute respiratory distress syndrome (ARDS). ARDS does not result directly from viral replication or infection, but rather from an immune system overreaction and an imbalance in inflammation triggered by the viral infection (10). During the immune response, the virus can co-evolve with the host, employing various mechanisms to regulate the inflammatory response. This adaptability allows the virus to develop in ways that evade the host cell’s defense mechanisms (11, 12). For instance, SARS-CoV-2, the virus that causes COVID-19, employs a variety of strategies to evade the immune system, including the manipulation of the innate immune response by reducing interferon (IFN) levels, as detailed in recent research. Patients with mild or moderate COVID-19 exhibit low levels of type I and type III IFNs in their sera (13). These findings highlight the significance of a dynamically balanced inflammatory response in the body, which exhibits antiviral properties. However, the specific mechanisms governing this delicate balance during EV71 infection remain unclear and warrant further investigation.

The activation of pyroptosis involves two distinct pathways: the classic pathway, dependent on Caspase-1, and the non-canonical pathway, dependent on Caspase-11, driven by different inflammatory caspases (14). In the classic pathway, Caspase-1 activation leads to the secretion of pro-inflammatory cytokines, including IL-1β and IL-18, into the extracellular space, thereby intensifying the inflammatory response (15). Caspase-1 specifically cleaves Gasdermin-D, releasing its N-terminal domain (16). The released N-terminal domain of Gasdermin-D binds to phospholipid molecules on the cell membrane, forming pores. These pores disrupt the osmotic balance of the cell, leading to swelling and the eventual rupture of the cell membrane, which results in pyroptotic cell death (17, 18). The non-canonical pathway, dependent on Caspase-11, encompasses critical signaling molecules, including Gasdermin-D, Caspase-1, IL-1β, and IL-18. Upon stimulation by viral and other signals, activated Caspase-11 mediates cleavage of Gasdermin-D, thereby generating its N-terminal effector domain. This cleavage induces perforation and rupture of the cell membrane, releasing cellular contents and triggering an inflammatory response. Additionally, activated Caspase-11 can activate NLRP3/Caspase-1, leading to the cleavage of IL-1β and IL-18 and further initiating an inflammatory response (19, 20). These pathways elucidate the complex mechanisms through which Caspase-1 and Caspase-11 orchestrate pyroptosis, emphasizing their critical roles in the secretion of pro-inflammatory cytokines and the induction of cell membrane rupture, inflammatory cytokines, and inducing cell membrane disruption, ultimately contributing to the inflammatory response.

In the context of EV71 virus infection, the inflammatory response is pivotal not only to clearing the virus but also in preventing extensive tissue damage, as evidenced by studies linking EV71 to systemic inflammatory response syndrome (SIRS) and its potential to cause severe complications. Regulation of inflammatory signaling pathways is critical to maintaining the balance and stability of the host’s internal environment (21). To investigate the pathogenesis of EV71-induced brain injury, we established an EV71 infection mouse model with Caspase-1/Caspase-11 blockade. This model enabled us to examine the interplay between the Caspase-1-dependent canonical pyroptosis pathway and the Caspase-11-dependent non-canonical pathway, offering a novel regulatory perspective to elucidate the immune mechanisms underlying EV71 infection.

## MATERIALS AND METHODS

### Cells and viruses

Human malignant embryonal rhabdomyosarcoma cells (RD) were cultured in DMEM medium (Nissui, Tokyo, Japan) containing the following components: heat-inactivated fetal bovine serum (FBS; Gibco, New York, USA), 100 U/mL penicillin, and 100 µg /mL streptomycin (Gibco, New York, USA). Cells were cultured at 37°C and 5% CO_2_.

EV71 (Genebank serial number: EU703812) virus strain was inoculated in RD cells. The Reed-Muench method was used to calculate the TCID_50_ (tissue culture infectious dose 50%) of EV71 virus at 48 h.

### Animals

BALB/c mice weighing 18–22 g in this study were obtained from Changchun Yisi Experimental Animal Technology Co., Ltd. (SCXK (JI)−2020-0002; Changchun, China). Mice were housed in a temperature-controlled room (12 h dark/light cycle, 21–25°C, and 55 ± 5% relative humidity) and were provided with adequate food and water. Before the start of the experiment, the mice were allowed to acclimate to the environment for one week. During this period, BALB/c mice were housed in groups with a 2:1 male-to-female ratio. Pregnant female mice were placed individually in cages to await delivery. EV71 virus concentrate (25 µL/g) was used to infect 1-day-old BALB/c suckling mice via the intraperitoneal (ip) route, and the injections were given continuously for 3 days. Five time points, namely, days 3, 5, 7, 10, and 14, were selected to detect the expression of inflammatory proteins. Four hours prior to infection in suckling mice, the following were administered via ip: VX765 (50 µg/g, Selleckchem, USA) or Wedelolactone (30 µg/g, MCE, USA), both dissolved and diluted in DMEM medium; the control group received an equal volume of DMEM (the solvent used for diluting the inhibitors). The experiment on the infection of suckling mice was conducted under Animal Biosafety Level 2 conditions. All animals were managed following the Guide for the Care and Use of Laboratory Animals of the National Institutes of Health, and the Animal Care and Use Committee of Jiamusi University approved all the procedures.

### Antibodies

The main antibodies used in this study were as follows: against mouse EV71 VP-1 (Abnova, Cat.No.: MAB1255-MOB Heidelberg, Germany); β-actin(ZSGB-BIO, Cat.No.: AW0505 Beijing, China); IL-1β (Abcam, Cat.No.: ab283818 Cambridge, UK); Caspase-11 (Abcam, Cat.No.: ab214185 Cambridge, UK); IL-18 (Affinity, Cat.No.: DF6252 Ohio, USA); Caspase-1 (Abcam, Cat.No.: ab179515 Cambridge, UK); GSDMD (Santa Cruz, Cat.No.: Sc-393581 California, USA); Cleaved-GSDMD (Affinity, Cat.No.: AF4012 Ohio, USA); NeuN (Servicebio, Cat.No.: GB11138); GFAP (Servicebio, Cat.No.: GB11096); and Myelin (Servicebio, Cat.No.: GB12226). Secondary antibodies included the following antibodies: Alexa Fluor 488 goat anti-rabbit IgG, Alexa Fluor Cy3 anti-mouse IgG, horseradish peroxidase (HRP)-conjugated anti-rat IgG, HRP-conjugated anti-mouse IgG (the above antibodies were purchased from Shanghai Beyotime Biotechnology Co., Ltd., China.)

### Cytopathic effects

RD cells were cultured in DMEM medium containing 2% FBS at 37℃ and 5% CO_2_. When the cells adhered to 80% of the surface and grew to 80% confluence, the cells were rinsed once with PBS, and 100 TCID_50_ of EV71 virus was inoculated. The effect of the EV71 virus on the growth status of RD cells was observed at 24 h, 48 h, and 72 h.

### Clinical symptom score

After EV71-infected BALB/c suckling mice, the weight and disease score of the suckling mice were recorded every day. The disease score evaluation method was as follows (Table 1):

**TABLE 1:** Clinical disease scores of BALB/c suckling mice

Clinical disease score	Clinical presentation
0	Healthy
1	Reduced activity
2	Weight loss or emaciation
3	Unilateral hind limb muscle paralysis
4	Bilateral hind limb muscle paralysis
5	Near death or death

### Histopathology

Samples were embedded in paraffin, cut into 4 µm sections, and stained with hematoxylin and eosin (H&E) for histological evaluation. The sectioned slides were immersed in a hematoxylin staining solution and stained for a specific period to highlight the cell nuclei and other cellular nucleic acid components. The slides were then gently rinsed to remove any excess dye, followed by immersion in eosin staining solution for a specific period to highlight the cytoplasm and cell granules. After rinsing gently, the slides were mounted with neutral resin, and the morphological changes of mouse brain tissue were observed under a microscope.

### Immunofluorescence staining

Tissue samples were mounted on slides in paraffin blocks (4 µm sections), deparaffinized five times in xylene for 15 min each, and rehydrated in an ethanol gradient (95%, 70%, 50%, and 30%). Hydrogen peroxide and 10 mM citrate buffer (pH 6.0) were used for antigen retrieval. Nonspecific peroxidase activity was blocked with 30% bovine serum albumin (BSA) for 30 min. *Cycle 1 (iF488-TSA): *The primary antibody (1:100) was incubated overnight in a wet chamber at 4℃. An enzyme-labeled secondary antibody (1:200) was used, followed by iF488-TSA amplification. *Cycles 2–3 (IF555/iF647-TSA): *(IF555-TSA) and (iF647-TSA) staining were performed by repeating steps 4–6, with a stripping step between cycles. The sections were stained with DAPI, treated with a self-quenching fluorescence agent, and then sealed with an anti-quenching fluorescence mounting agent (Servicebio, Cat.No.: G1226-100T Wuhan, China). The co-localization was quantified using the Pearson correlation coefficient and Mander’s overlap in ImageJ.

### Western blot analysis

After the tissue was lysed in radioimmunoprecipitation assay buffer containing a protease inhibitor cocktail for 20 min, the protein mixture was centrifuged at 13,000 rpm for 15 min at 4°C to obtain the supernatant. The supernatant was boiled with a corresponding volume of 5× sodium dodecyl sulfate (SDS) loading buffer, then fractionated by SDS-polyacrylamide gel electrophoresis (SDS-PAGE), and transferred to a polyvinylidene difluoride membrane. After the membrane was blocked with 5% skim milk powder for 1 h at room temperature, the membrane was incubated with primary antibodies (1:1,000) overnight at 4°C, followed by incubation with secondary antibodies (Beyotime) at the corresponding concentration (1:5,000), and finally detected by chemiluminescence.

### Statistical analysis

Experimental data were expressed as mean ± SD from at least three independent experiments. One-way ANOVA was used to compare the three groups, followed by post hoc multiple comparisons using the Student–Newman–Keuls test. The Mantel-Cox log-rank test was used to assess survival rates. *P* values < 0.05 were considered statistically significant.

## RESULTS

### EV71 induces an inflammatory response in the brain tissue of BALB/c suckling mice

Using the Reed-Muench method (22), we determined the 72-hour tissue culture infectious dose (TCID_50_) of the EV71 virus, with a final value of 10^-4.12^/mL. Following EV71 infection of RD cells, distinct cytopathic effects (CPE) were observed over time. At 24 h post-infection (hpi), RD cells underwent a clear morphological change: they shifted from their typical spindle-like shape to rounded, bead-like structures and appeared in a scattered distribution. As the infection duration extended to 48 and 72 hpi, the proportion of cells exhibiting CPE increased markedly (Fig. S1). This progression was accompanied by pronounced cellular necrosis, and the affected cells eventually detached from the surface of the culture vessel.

**Fig 1:**
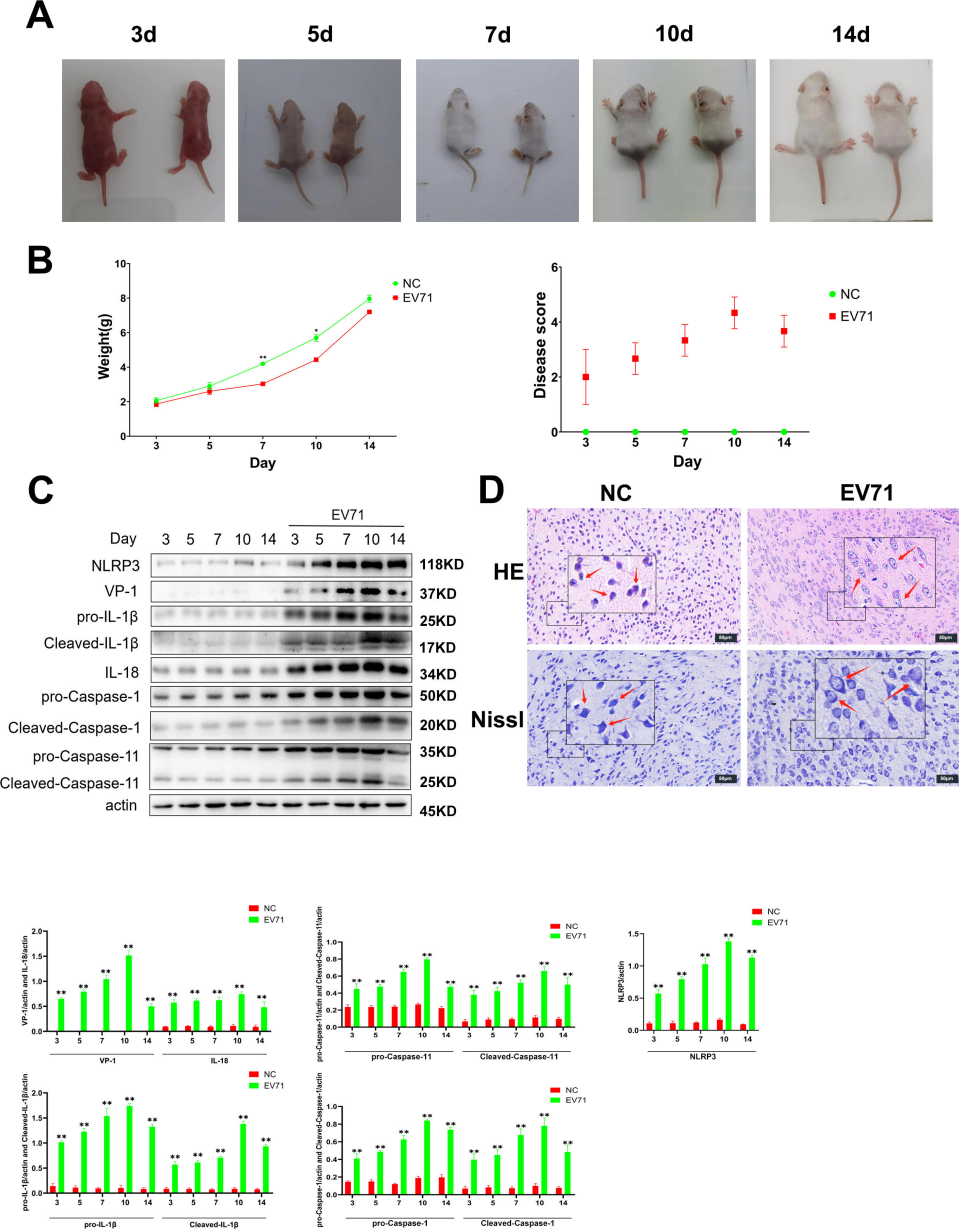
EV71 induces an inflammatory response in the brain tissue of BALB/c suckling mice (**A**). Appearance of BALB/c suckling mice: normal mice are shown on the left and EV71-infected mice on the right. (**B**) The weight gain of EV71-infected suckling mice gradually decreases with the prolongation of infection time. The clinical disease score of EV71-infected suckling mice increases with the prolongation of infection time. (**C**) Expression of NLRP3, VP-1, pro-IL-1β, cleaved-IL-1β, IL-18, pro-Caspase-1, Cleaved-Caspase-1, pro-Caspase-11, and Cleaved-Caspase-11 proteins in the brain tissue of BALB/c suckling mice. EV71 induces an inflammatory response in the brain tissue of BALB/c suckling mice (30 µg per lane). (**D**) H&E staining 10 days after infection. The red arrow indicates that the number of neurons is significantly reduced; a large number of neurons are necrotic; and the tissue is cribriform softening lesions (scale: 50 µm). Nissl staining was performed 10 days after infection. The arrow indicates the disintegration of the central Nissl bodies within the cytoplasm, characterized by dissolution and disappearance (scale: 60 µm). NC: normal control group; EV71: enterovirus 71 infection group. Compared with NC group: *P < 0.05, **P < 0.01.

Compared with the control, those BALB/c suckling mice infected with EV71 exhibited marked neurological deterioration. In the early stages of infection, affected mice showed progressive weight loss, lethargy, hind limb weakness, hunched posture, and decreased fur density (Fig. 1A). As the infection advanced, some mice succumbed during the middle and late stages. Prolonged EV71 infection was associated with persistent body weight loss, which correlated with a time-dependent increase in clinical symptom scores (Fig. 1B). VP1, the major structural and immunodominant capsid protein of EV71, was undetectable in the brain tissue of uninfected mice but was abundantly expressed following infection, with levels peaking on the tenth day post-infection and paralleling viral proliferation. Notably, EV71 infection led to the activation of the NLRP3 inflammasome and the subsequent upregulation of IL-1β and IL-18. The concentrations of IL-1β and IL-18 in brain tissue of infected mice increased significantly throughout infection, also reaching their maximum on day 10 (Fig. 1C). Therefore, brain tissue samples from day 10 post-infection were used for subsequent experiments.

Hematoxylin and eosin (H&E) staining revealed a marked decrease in neuronal density in the brain tissue of BALB/c suckling mice at day 10 following EV71 infection. Numerous neurons exhibited features of necrosis, and the brain parenchyma displayed prominent cribriform lesions, foci of tissue softening (encephalomalacia), neuronal edema, and conspicuous cytoplasmic vacuolation. These pathological changes underscore the potent ability of EV71 to elicit inflammatory injury within the central nervous system of suckling mice. Consistent with these findings, Nissl staining demonstrated pronounced neuronal swelling in infected tissues at day 10 post-infection, accompanied by disintegration or dissolution of central Nissl bodies in the cytoplasm. Remnants of Nissl substance were often observed distributed around the periphery of neurons, appearing as a pale or grayish background (Fig. 1D).

### EV71 infection induces selective neuronal pyroptosis in the mouse brain

**Fig 2:**
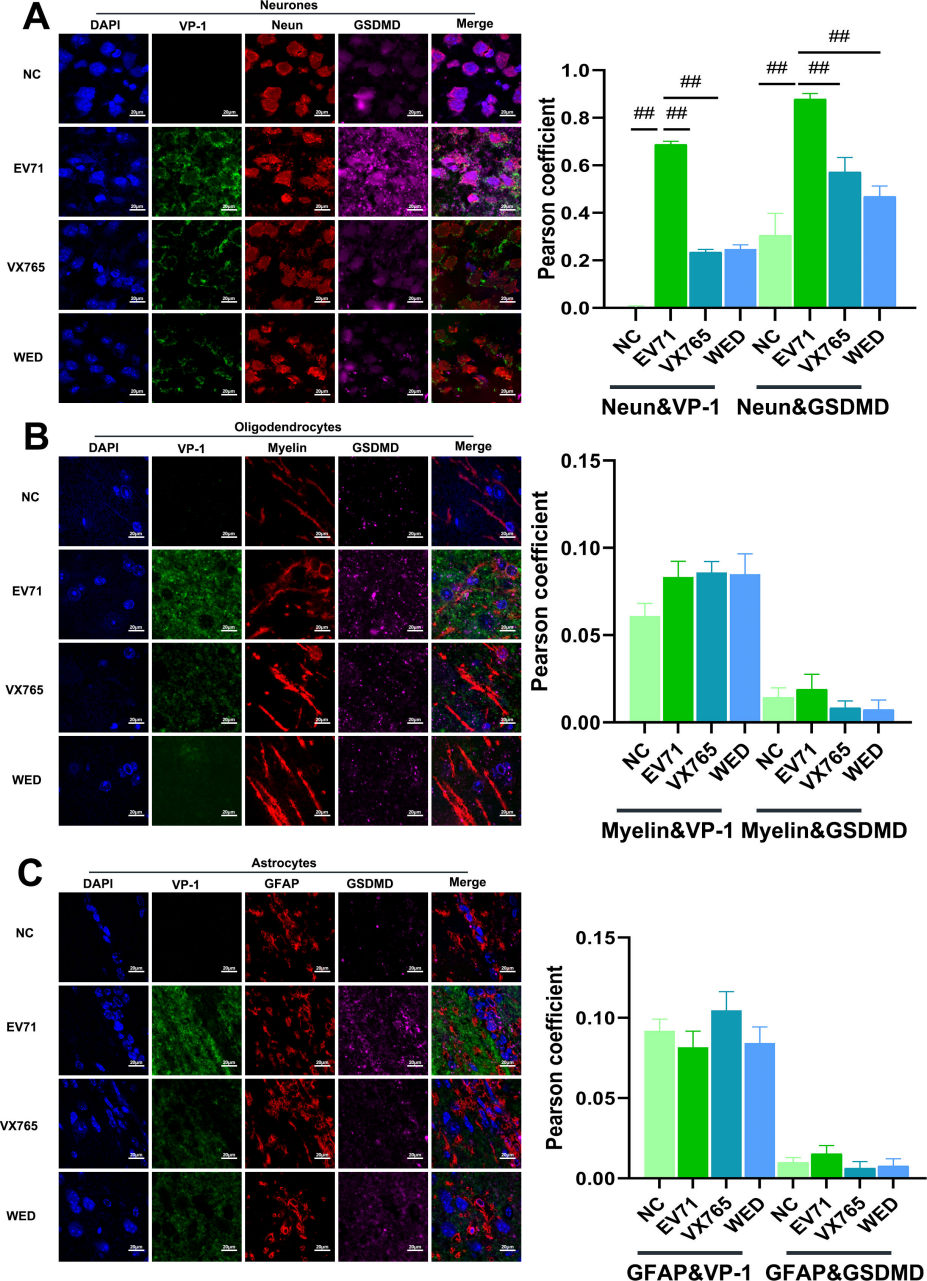
EV71 infection primarily induces pyroptosis in cerebral neurons rather than neuroglial cells. (**A**). Neurons: quadruple staining confocal image showing DAPI (blue), VP-1 (green), NeuN (red), and GSDMD (magenta) in neurons. (**B**) Oligodendrocytes: quadruple staining confocal image showing DAPI (blue), VP-1 (green), Myelin (red), and GSDMD (magenta) in oligodendrocytes. (**C**) Astrocytes: quadruple staining confocal image showing DAPI (blue), VP-1 (green), GFAP (red), and GSDMD (magenta) in astrocytes. Compared with EV71 ##*P* < 0.01 (scale: 25 µm).

Quadruple immunofluorescence staining demonstrated a distinct pattern of VP-1 and NeuN co-localization across experimental groups (Fig. 2). Following EV71 infection, the co-localization rates of VP-1, GSDMD, and the neuronal marker NeuN increased significantly (Fig. 2A). By contrast, the co-localization of VP-1 and GSDMD with Myelin (an oligodendrocyte marker, Fig. 2B) and GFAP (an astrocyte marker, Fig. 2C) remained comparatively low. These findings indicate that EV71 infection primarily targets neurons and induces pyroptosis within neuronal populations. Treatment with VX765 and Wedelolactone reduced the extent of VP-1/NeuN/GSDMD co-localization, suggesting that inhibition of Caspase-1 and Caspase-11 pathways can attenuate EV71-induced neuronal pyroptosis. Notably, triple-label analysis further confirmed that the observed co-localization of VP-1, NeuN, and GSDMD within neurons is closely associated with the occurrence of pyroptosis following EV71 infection (Fig. 2A).

### Selective inhibition of Caspase-1 or Caspase-11 attenuates EV71-induced neuroinflammation and brain injury

**Fig 3:**
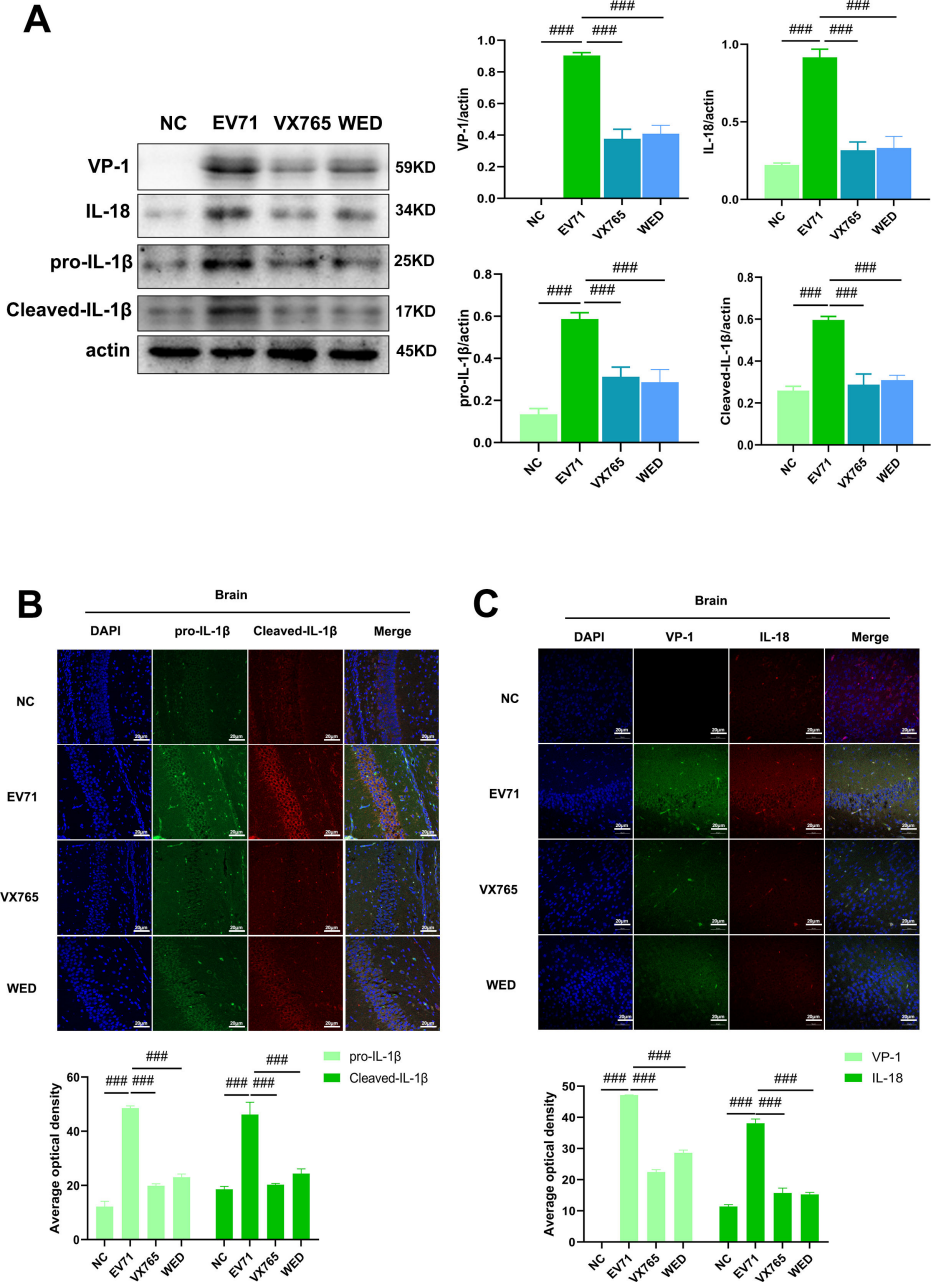
EV71 induces the activation of inflammatory responses in the brain tissue of BALB/c suckling mice by activating the Caspase-1 and Caspase-11 pathways. (**A**) Western blotting of VP-1, pro-IL-1β, cleaved-IL-1β, and IL-18 proteins in the brain tissue of suckling mice (30 µg per lane). (**B**) Co-localization staining of pro-IL-1β and cleaved-IL-1β in the brain tissue of suckling mice. (**C**) Co-localization staining of VP-1 and IL-18 in the brain tissue of suckling mice. NC: normal control group; EV71: enterovirus 71 infection group; VX765: EV71 infection group treated with VX765; WED: EV71 infection group treated with Wedelolactone. Compared with the EV71 group: ###*P* < 0.001 (scale bar: 50 µm).

**Fig 4:**
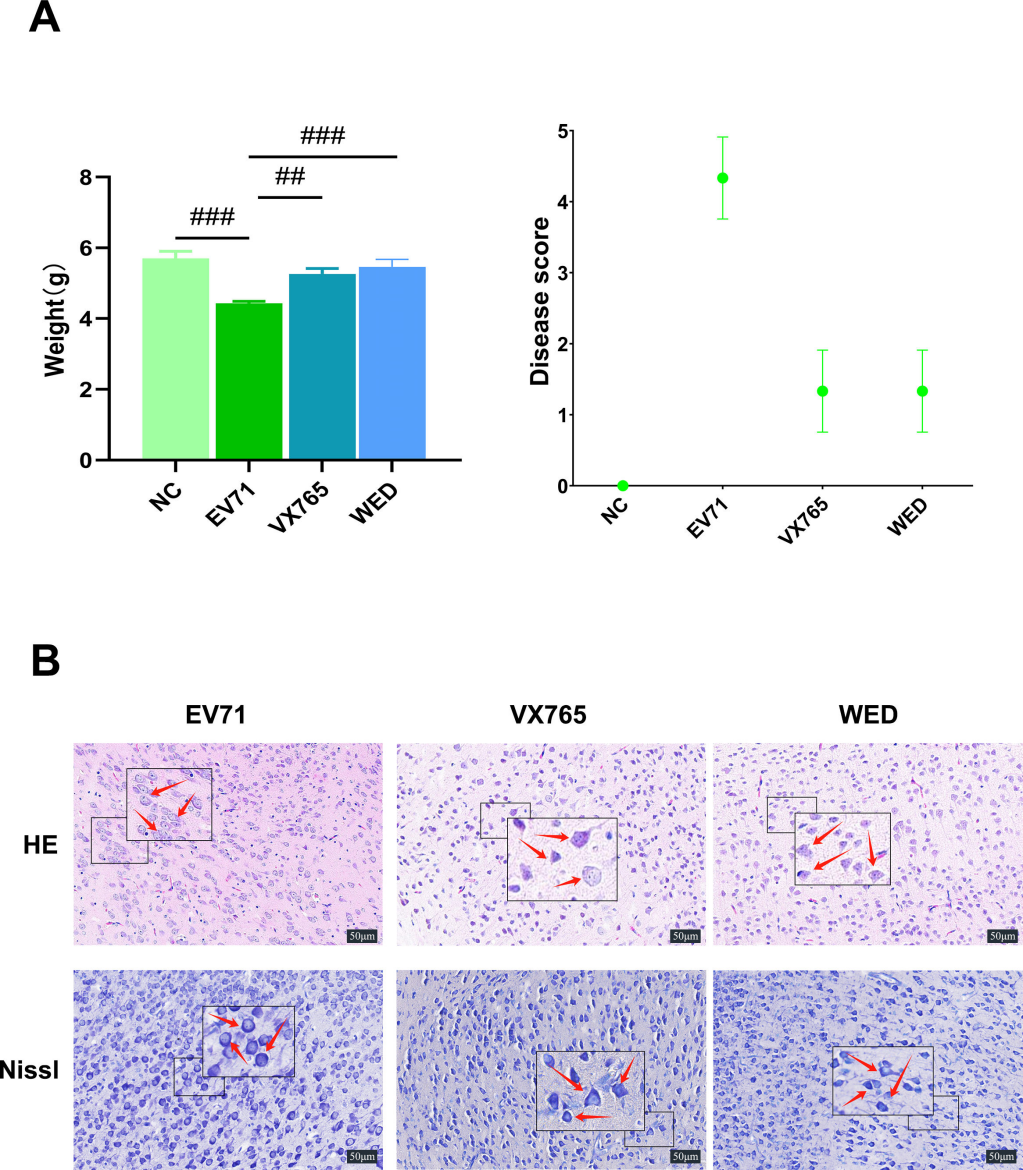
Blocking Caspase-1 and Caspase-11 pathways can reduce the pathogenicity of EV71 in BALB/c suckling mice. (**A**) Caspase-1 and Caspase-11 inhibitors attenuate EV71 pathogenicity. The weight loss of EV71-infected suckling mice was restored under the treatment of Caspase-1 and Caspase-11 inhibitors compared with the EV71-infected group, and the clinical disease scores basically returned to normal. (**B**) The NC group showed normal performance after H&E staining. The red arrows in the EV71 infection group indicate a significant reduction in the number of neurons, a large number of neuronal necrosis, and cribriform softening of the tissue. These lesions were significantly improved after the use of Caspase-1 and Caspase-11 inhibitors. The results of Nissl staining showed that the pathological injury of the brain tissue of BALB/c mice induced by EV71 infection was significantly reduced by Caspase-1 and Caspase-11 inhibitors, the swelling of neurons was restored, the Nissl bodies were polygonal, and the nucleus was located in the center. Compared with the EV71 infection group: ##*P* < 0.01, ###*P* < 0.001.

Treatment with the Caspase-1 inhibitor VX765 and the Caspase-11 inhibitor Wedelolactone markedly suppressed activation of their respective targets, resulting in a significant reduction in the levels of pro-IL-1β, cleaved IL-1β, and IL-18 in the tissues of EV71-infected suckling mice (Fig. 3). This decrease reflects an effective attenuation of the inflammatory response. Additionally, both VX765 and Wedelolactone led to a substantial reduction in VP-1 expression in the brain tissue of infected mice, accompanied by significant increases in body weight and lower clinical disease scores (Fig. 4A). Furthermore, these treatments effectively alleviated brain tissue damage in suckling mice. HE staining showed that the treatment with Caspase-1 and Caspase-11 inhibitors significantly alleviated the pathological damage of brain tissue and reduced neuronal necrosis in BALB/c mice infected with EV71. The results of Nissl staining showed that the pathological damage of brain tissue in BALB/c mice induced by EV71 infection was significantly alleviated after treatment with Caspase-1 and Caspase-11 inhibitors, and the swelling of neuronal cells was recovered. Nissl bodies were polygonal, and the nucleus was located in the center (Fig. 4B). Collectively, these findings provide compelling, multi-dimensional evidence that selective inhibition of Caspase-1 and Caspase-11 not only suppresses EV71-induced neuroinflammation in a targeted manner but also exerts robust protective effects on brain tissue, with this dual outcome primarily mediated by blocking the maturation and extracellular release of the pro-inflammatory cytokines IL-1β and IL-18—an effect that disrupts the “viral infection-inflammation-pyroptosis” pathogenic cascade underlying EV71-associated neurological injury.

### Cross-talk between Caspase-1 and Caspase-11 signaling drives pyroptosis during EV71 infection

EV71 infection triggers a robust inflammatory response via the activation of IL-1β and IL-18, which is mediated by two main pyroptotic pathways: the classical Caspase-1-dependent pathway and the non-classical Caspase-11-dependent pathway. In brain tissue of EV71-infected suckling mice, we observed a marked upregulation of pro-Caspase-1, cleaved Caspase-1, pro-Caspase-11, cleaved Caspase-11, and GSDMD. Administration of the Caspase-1 inhibitor VX765 or the Caspase-11 inhibitor Wedelolactone resulted in significant decreases in the expression of these proteins. These results were substantiated by colocalization analyses of Caspase-1, Caspase-11, and GSDMD, which were consistent with the Western blot findings.

**Fig 5:**
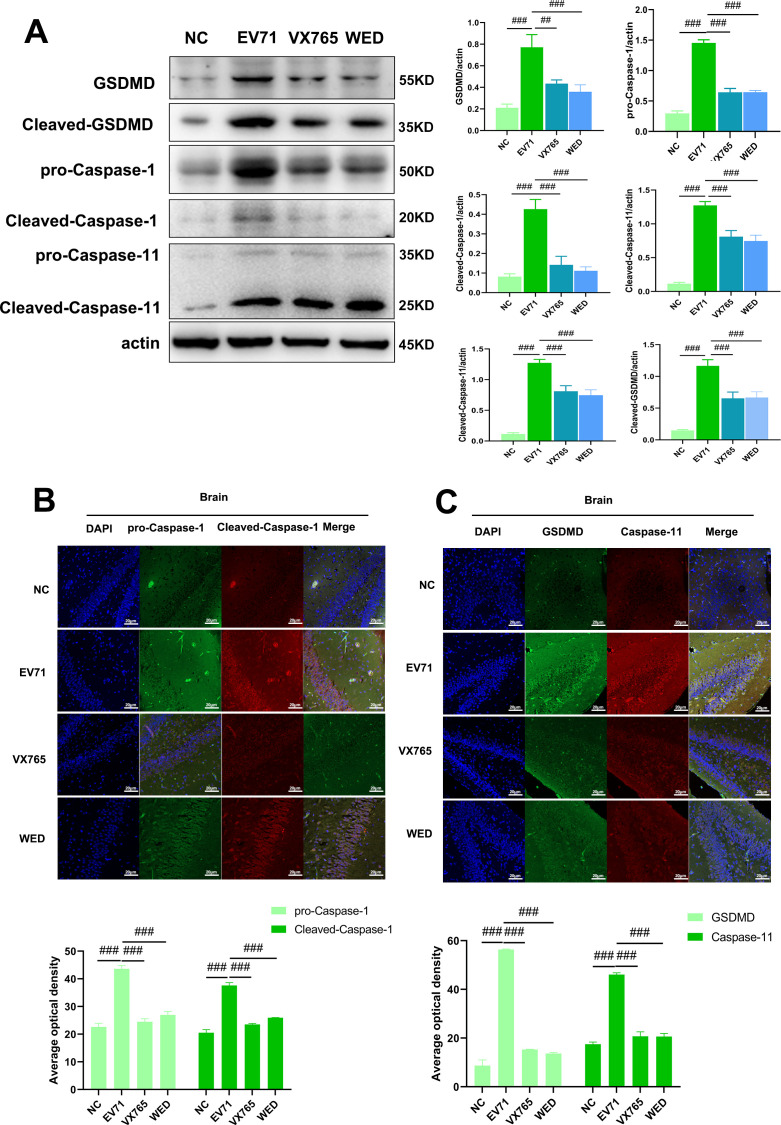
Caspase-1 and Caspase-11 pathways jointly regulate EV71-induced pyroptosis. (**A**) Western blot of GSDMD, Caspase-11, and Caspase-1 signaling pathway proteins in the brain tissue of suckling mice (30 µg per lane). (**B**) Co-localization staining of pro-Caspase-1, cleaved-Caspase-1, in the brain tissue of suckling mice. (**C**) Co-localization staining of GSDMD, Caspase-11, in the brain tissue of suckling mice. NC: normal control group; EV71: enterovirus 71 infection group; VX765: EV71 infection group treated with VX765; WED: EV71 infection group treated with Wedelolactone (scale bar: 50 µm). Compared with the EV71 infection group: ##*P* < 0.01, ###*P* < 0.001.

Notably, inhibition of either the Caspase-1 or Caspase-11 pathway led to a concomitant downregulation of the other pathway. Specifically, blockade of Caspase-1 suppressed Caspase-11 expression and vice versa. In parallel, GSDMD expression in brain tissue was dramatically reduced following inhibition of either pathway (Fig. 5). These findings demonstrate a cross-regulatory interaction between the Caspase-1 and Caspase-11 pathways, indicating that inhibition of one pathway is sufficient to suppress the activity of both, ultimately attenuating pyroptosis. This newly identified reciprocal regulation between the two pathways may represent a critical mechanism for controlling pyroptotic cell death during EV71 infection.

## DISCUSSION

EV71, a significant cause of hand-foot-mouth disease, predominantly impacts children under 5 years of age. While most infections are self-limiting, severe cases can result in neurological complications, such as aseptic meningitis, acute flaccid paralysis, and brainstem encephalitis (6, 23). In the present study, a neonatal mouse model of EV71 infection was established, displaying clinically relevant symptoms including weight loss, drowsiness, and limb weakness. Histopathological analysis revealed significant neuronal loss, necrosis, sieve-like softening foci, and dissolution of Nissl bodies, consistent with the clinical neuropathology.

EV71 encodes four structural proteins (VP-1, VP-2, VP-3, and VP-4), among which VP-1 plays a pivotal role in immune evasion, viral proliferation, and disease progression (24, 25). Our study confirmed increased VP-1 expression in brain tissue following intraperitoneal infection, as demonstrated by Western blot and immunofluorescence analyses, reinforcing the suitability of this model for investigating EV71 neuropathogenesis.

Viral infection triggers a rapid host immune response, characterized primarily by inflammation aimed at containing the spread of the virus (26–28). In severe infections, excessive activation of signaling pathways leads to a cytokine storm, exacerbating tissue injury (29, 30). The inflammasome, particularly the NLRP3 complex, acts as a critical mediator of this response by activating Caspase-1, which in turn processes IL-1β and IL-18—key pro-inflammatory cytokines that drive pyroptotic cell death (31–33). In our model, EV71 infection resulted in elevated levels of pro-IL-1β, cleaved IL-1β, and IL-18 in brain tissue, peaking on day 10 post-infection. This time point was chosen for mechanistic studies, as it represented the maximal inflammatory response.

Pyroptosis, a lytic and pro-inflammatory form of cell death, is now recognized as the principal mechanism underlying the secretion of IL-1β and IL-18 in response to microbial infection (34, 35). Caspase-1 and Caspase-11 are central mediators of pyroptosis, but they have distinct yet overlapping roles. Caspase-1 directly cleaves pro-IL-1β and pro-IL-18, whereas Caspase-11 is required for non-canonical inflammasome activation and effective pyroptosis in response to certain pathogens, as well as for GSDMD cleavage and pore formation (36, 37). EV71 can induce inflammatory responses through multiple pathways. For instance, its structural proteins (e.g., VP1) and non-structural proteins (e.g., 2A and 3C proteases) can activate Toll-like receptors (TLRs) or retinoic acid-inducible gene I (RIG-I)-like receptors (RLRs), thereby triggering NF-κB/MAPK signaling cascades. This activation ultimately leads to the induction of pro-inflammatory cytokines (e.g., TNF-α, IL-6) in an inflammasome-independent manner (38). Although EV71 elicits inflammation via diverse routes, our findings demonstrate that the Caspase-1/Caspase-11-gasdermin D (GSDMD) axis is critical for mediating IL-1β/IL-18-dependent brain injury. This highlights the axis as a promising therapeutic target for EV71-associated neurological complications. In subsequent studies, we will further investigate the mechanisms underlying EV71 infection-induced inflammatory responses.

Our results demonstrated that EV71 infection in the brain tissue of neonatal mice significantly upregulated both the pro-forms and cleaved forms of Caspase-1, Caspase-11, and GSDMD. Treatment with specific inhibitors (VX765 for Caspase-1 and Wedelolactone for Caspase-11) reduced these molecular markers and prevented pyroptotic damage, supporting that EV-A71-driven inflammasome activation and downstream pyroptosis are sensitive to inhibition of either caspase pathway and reinforcing our conclusion regarding their cross-regulation in EV71-mediated injury.

Wedelolactone has activities beyond Caspase-11 inhibition: prior studies indicate it suppresses NF-κB (via inhibiting IκBα phosphorylation/degradation) and modulates kinases such as Akt and MAPKs, which regulate inflammation and may indirectly affect pyroptosis (39). Nevertheless, the molecular mechanism of Caspase-11 activation suggests that it is dependent on oligomerization induced by direct LPS binding, a critical step independent of NF-κB transcriptional regulation (40). Notably, in this study, Wedelolactone exerted a potent inhibitory effect on Caspase-11, indirectly suggesting that it acted by interacting directly with Caspase-11 rather than indirectly regulating NF-κB. Future studies should complement NF-κB-related assays (such as IKK/IκBα phosphorylation, nuclear translocation of NF-κB p65) and integrate Caspase-11 knockdown cell models to elucidate whether the potential NF-κB regulation by Wedelolactone contributes to its Caspase-11 inhibition, and how this contributes to the full elucidation of its mechanisms. Specifically, Wedelolactone treatment was associated with reduced cleaved Caspase-11 (mirroring VX765’s effect on Caspase-1), along with corresponding decreases in GSDMD (the key pyroptosis executor) and attenuated pyroptotic damage. Importantly, our results demonstrate that pharmacological inhibition of either caspase led to a concomitant reduction in the activation of the other, as evidenced by decreased levels of cleaved Caspase-1, cleaved Caspase-11, and their shared downstream effector, cleaved GSDMD (Fig. 5). This interdependency could be interpreted through several non-mutually exclusive mechanisms. It may reflect an indirect, synergistic relationship within an amplified inflammatory signaling network, where the suppression of one pathway alleviates the overall inflammatory burden, thereby reducing the stimulus for the activation of the other. Alternatively, it might involve more direct, yet uncharacterized, regulatory crosstalk, such as the potential for Caspase-11 to upstream regulate the NLRP3/Caspase-1 axis (41), or the possibility that both pathways converge critically on GSDMD cleavage, creating a positive feedback loop that is disrupted when either arm is inhibited (42). Nevertheless, our findings robustly indicate that the pro-pyroptotic functions of Caspase-1 and Caspase-11 during EV71 infection are highly interdependent, and therapeutic targeting of either pathway can effectively disrupt this synergistic activation loop and mitigate pyroptotic brain injury.

Multiplex immunofluorescence further revealed that EV71 preferentially targets neurons rather than glial cells, as indicated by the robust colocalization of VP-1, NeuN, and GSDMD. Conversely, myelin and GFAP showed minimal co-localization, suggesting that glial cells are less susceptible to direct viral invasion, although they may still undergo secondary inflammatory injury. Notably, pharmacological inhibition of Caspase-1 or Caspase-11 reduced neuronal pyroptosis and viral load, resulting in alleviated tissue damage and improved clinical outcomes in infected mice.

Pyroptosis appears to play a dual role in the context of EV71 infection. In the early stage, it can be protective by restricting viral replication and spread (43, 44). However, excessive and sustained activation leads to overwhelming inflammation, neuronal loss, and severe neurological complications (10). The interaction between Caspase-1 and Caspase-11 amplifies this process, and disruption of either pathway is sufficient to prevent catastrophic tissue damage.

In summary, this study explored the mechanism of EV71-induced brain injury, focusing on pyroptosis-related pathways. In EV71-infected BALB/c suckling mice, we observed enhanced neuroinflammation (elevated IL-1β/IL-18) and upregulated Caspase-1, Caspase-11, and GSDMD in brain tissues. Pharmacological inhibition of Caspase-1 (VX765) or Caspase-11 (Wedelolactone) reduced pyroptosis-related molecule activation, IL-1β/IL-18 release, neuronal damage, and improved clinical symptoms. Importantly, our results suggest a potential interactive relationship between Caspase-1 and Caspase-11 during EV71 infection—suppressing one pathway was associated with reduced activation of the other, mitigating pyroptotic brain injury—though the specific nature of this interaction remains unclear.

**Fig 6:**
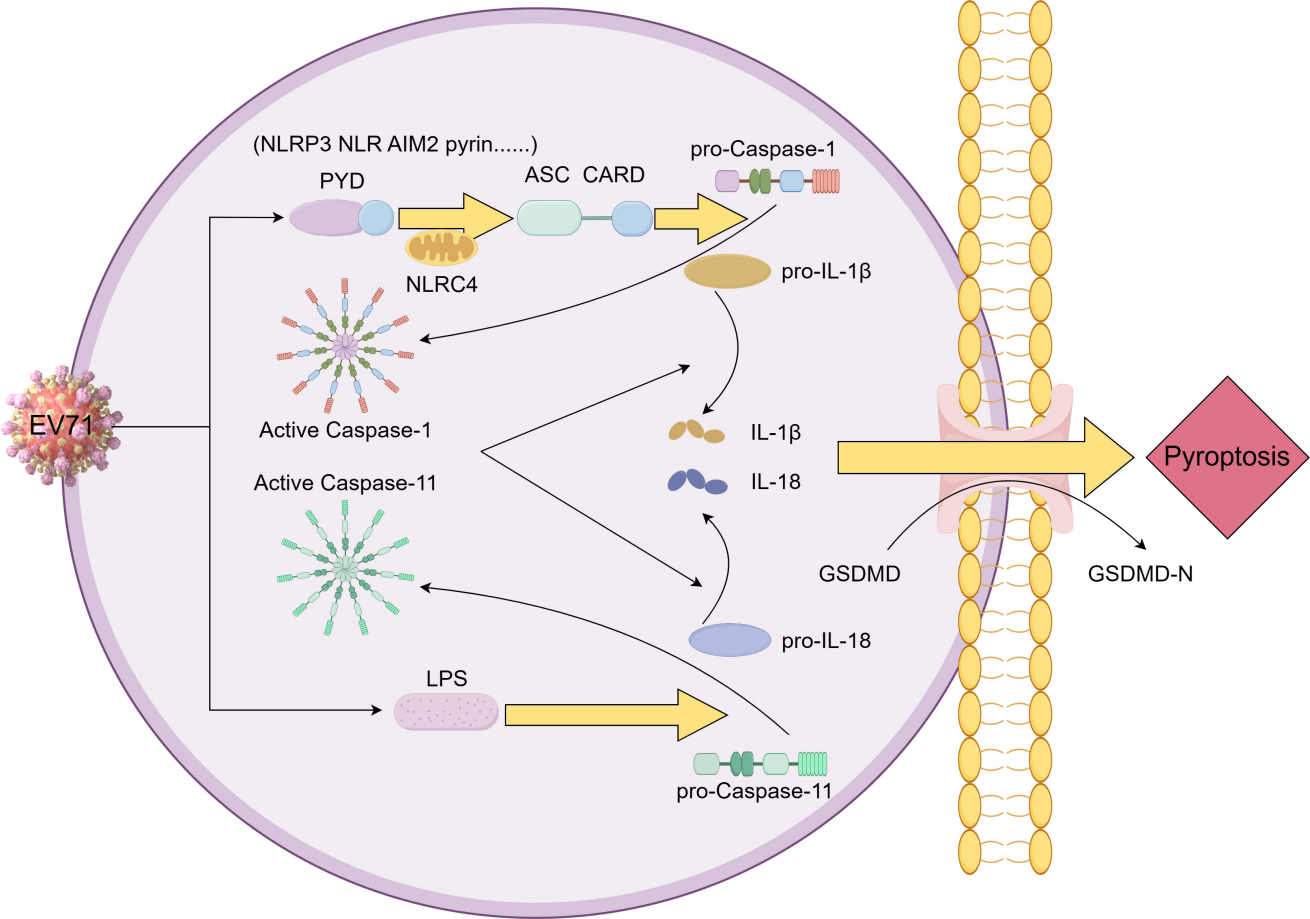
Diagram of the hypothesis of this study. After EV71 infection, the inflammasome is activated by a variety of microbial signals through ASC or NLRC4 adapters to activate Caspase-1. In addition, Caspase-11 can directly bind to and be activated by LPS. GSDMD forms pores in the plasma membrane, triggering the process of inflammatory pyroptosis. Activated Caspase-1 and Caspase-11 are able to cleave the precursors of IL-1β and IL-18 into mature forms and to tailor the linker between the N-terminus (gasdermin-N) and C-terminus (gasdermin-C) of GSDMD, breaking the self-inhibitory interaction. The released gasdermin-N structure binds to phosphatidylinositol in the plasma membrane and forms membrane pores with an inner diameter of about 12 to 14 nm. The formation of these pores disrupts cell permeability, leading to cell swelling and eventual rupture, further promoting the release of mature IL-1β and IL-18 from the cells.

Future studies will further investigate the regulatory mechanisms underlying Caspase-1/Caspase-11 crosstalk in EV71 infection (e.g., via gene knockout models) to verify and clarify their relationship, which may deepen understanding of EV71-associated neurological pathogenesis and inform targeted therapeutic strategies (Fig. 6).

## Data Availability

All data generated or analyzed during this study are included in this published article and its supplementary information files. All the data for this article can be accessed from https://www.scidb.cn/en/s/M3mIZn.

## SUPPLEMENTAL MATERIAL

Figure S1 jvi.01860-25-s0001.tif Morphological changes in RD cells infected with EV71.

Supplemental legend jvi.01860-25-s0002.docx Legend for Fig. S1.

ASM does not own the copyrights to Supplemental Material that may be linked to, or accessed through, an article. The authors have granted ASM a non-exclusive, world-wide license to publish the Supplemental Material files. Please contact the corresponding author directly for reuse.
