# Supplementary figures and images for: Enterovirus 71 infection induces pyroptotic brain injury via synergistic activation of classical inflammasome and viral gasdermin D cleavage
Source: J Virol. 2025 Nov 25;99(12):e01860-25. doi: 10.1128/jvi.01860-25 (PMC12724249; doi:10.1128/jvi.01860-25)

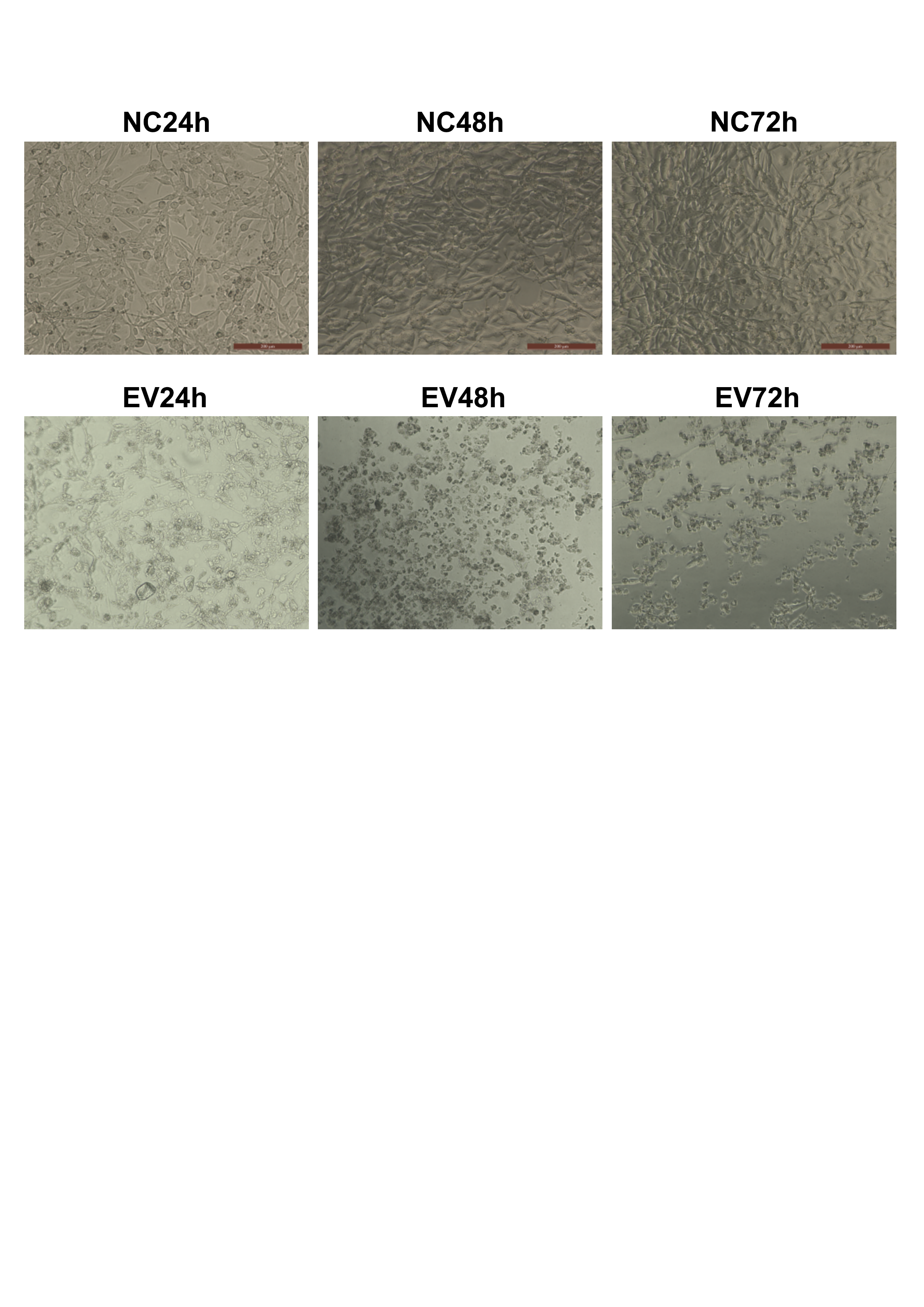

Supplement: Figure S1 — Morphological changes in RD cells infected with EV71. [file jvi.01860-25-s0001.tif]
