# Supplementary material for: Enterovirus 71 infection induces pyroptotic brain injury via synergistic activation of classical inflammasome and viral gasdermin D cleavage
Source: J Virol. 2025 Nov 25;99(12):e01860-25. doi: 10.1128/jvi.01860-25 (PMC12724249; doi:10.1128/jvi.01860-25)
Supplement: Supplemental legend — Legend for Fig. S1. [file jvi.01860-25-s0002.docx]

Supplementary Figure 1: Morphological changes in RD cells infected with EV71. Images show cytopathic effects (CPE) in EV71-infected cells compared to normal control (NC) at 24, 48, and 72 hours post-infection. (Scale bar: 200 μm).
